# Supplementary material for: ­­A high-throughput screen identifies inhibitors of the interaction between the oncogenic transcription factor ERG and the cofactor EWS
Source: PLoS One. 2020 Sep 11;15(9):e0238999. doi: 10.1371/journal.pone.0238999 (PMC7485968; doi:10.1371/journal.pone.0238999)

Figure 2A

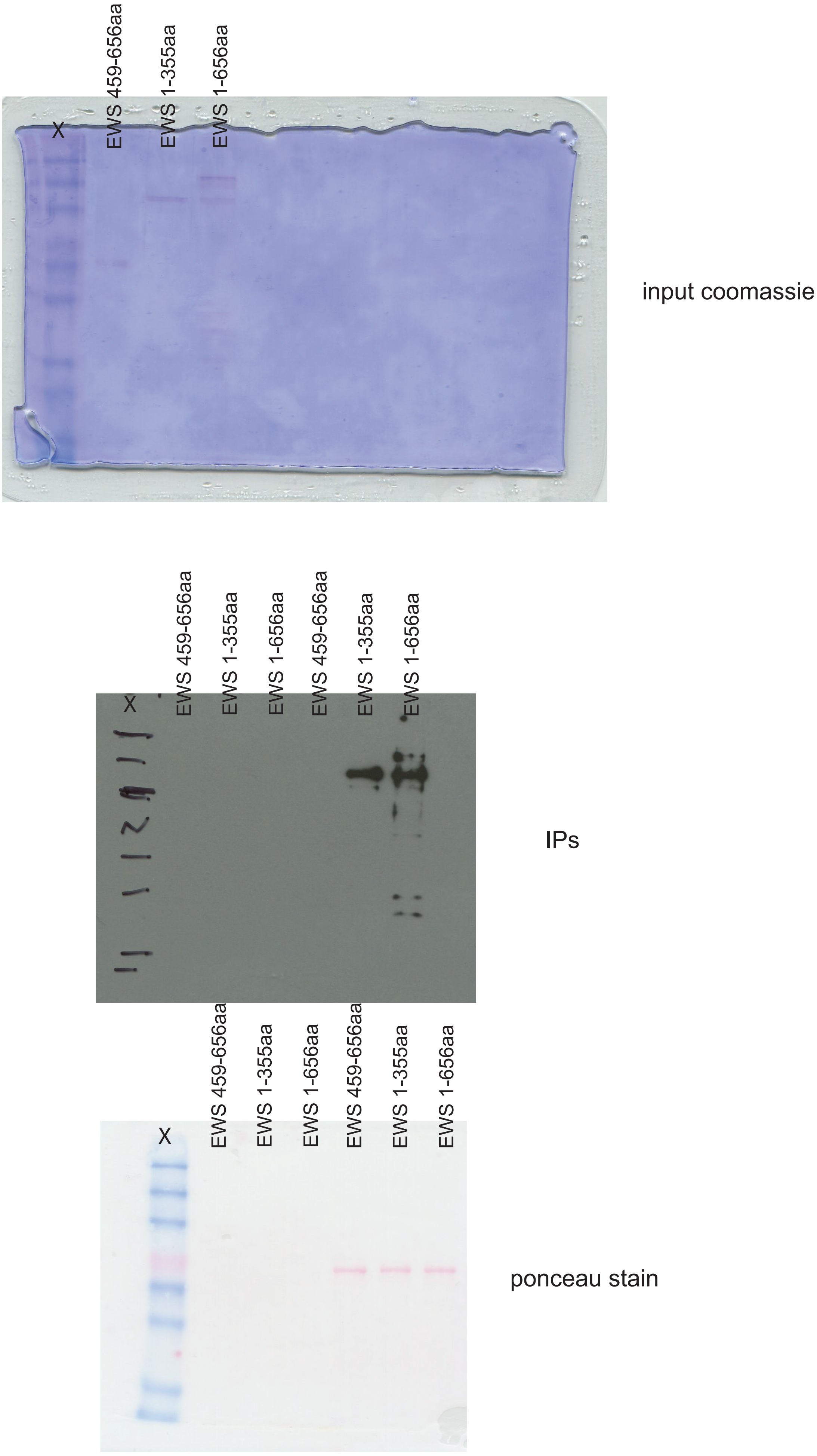

X
EWS 459-656aa
EWS 1-355aa
EWS 1-656aa
input coomassie
X
EWS 459-656aa
EWS 1-355aa
EWS 1-656aa
EWS 459-656aa
EWS 1-355aa
EWS 1-656aa
IPs
X
EWS 459-656aa
EWS 1-355aa
EWS 1-656aa
EWS 459-656aa
EWS 1-355aa
EWS 1-656aa
ponceau stain

Figure 5A

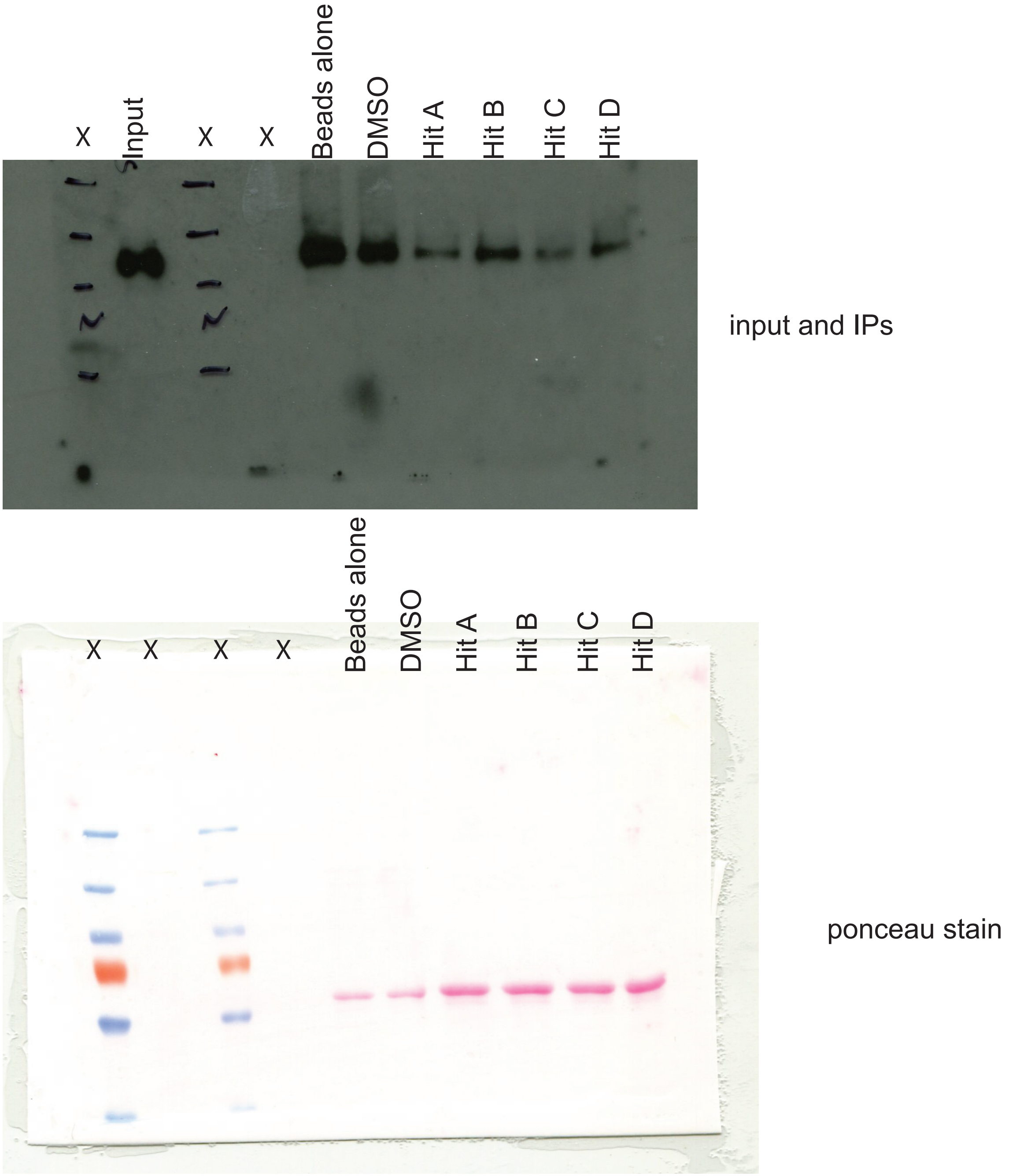

X
Input
X
X
Beads alone
DMSO
Hit A
Hit B
Hit C
Hit D
input and IPs
X
X
X
X
Beads alone
DMSO
Hit A
Hit B
Hit C
Hit D
ponceau stain

Figure 5B

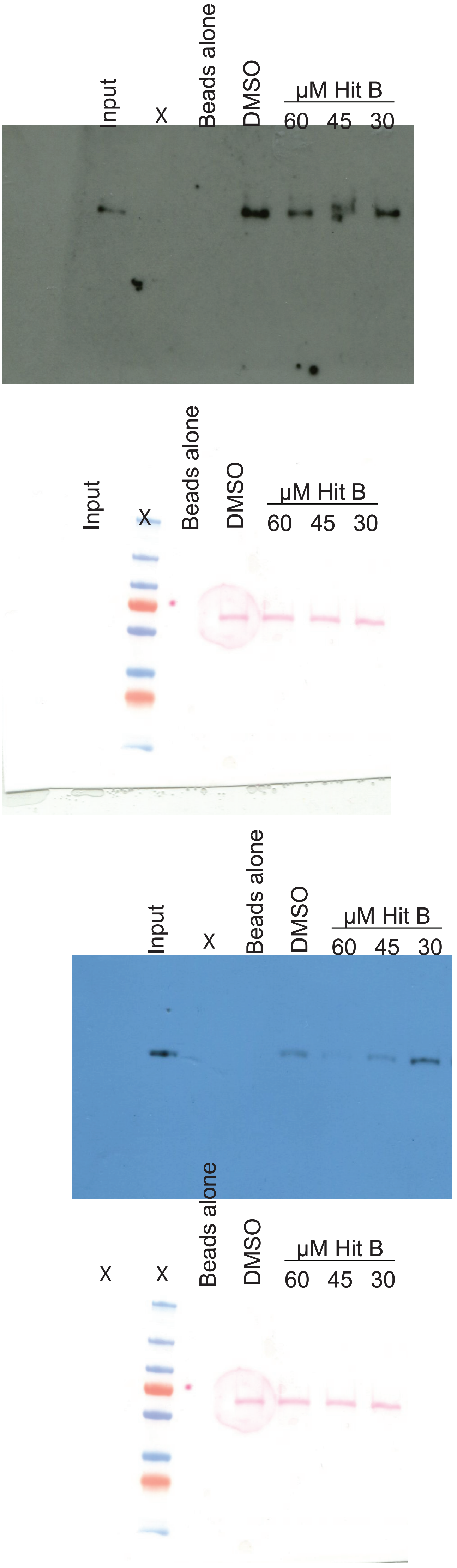
EXP1
Input
X
Beads alone
DMSO
µM Hit B
60 45 30
input and IPs
ponceau stain
EXP2
X
input and IPs
ponceau stain

Figure 5H

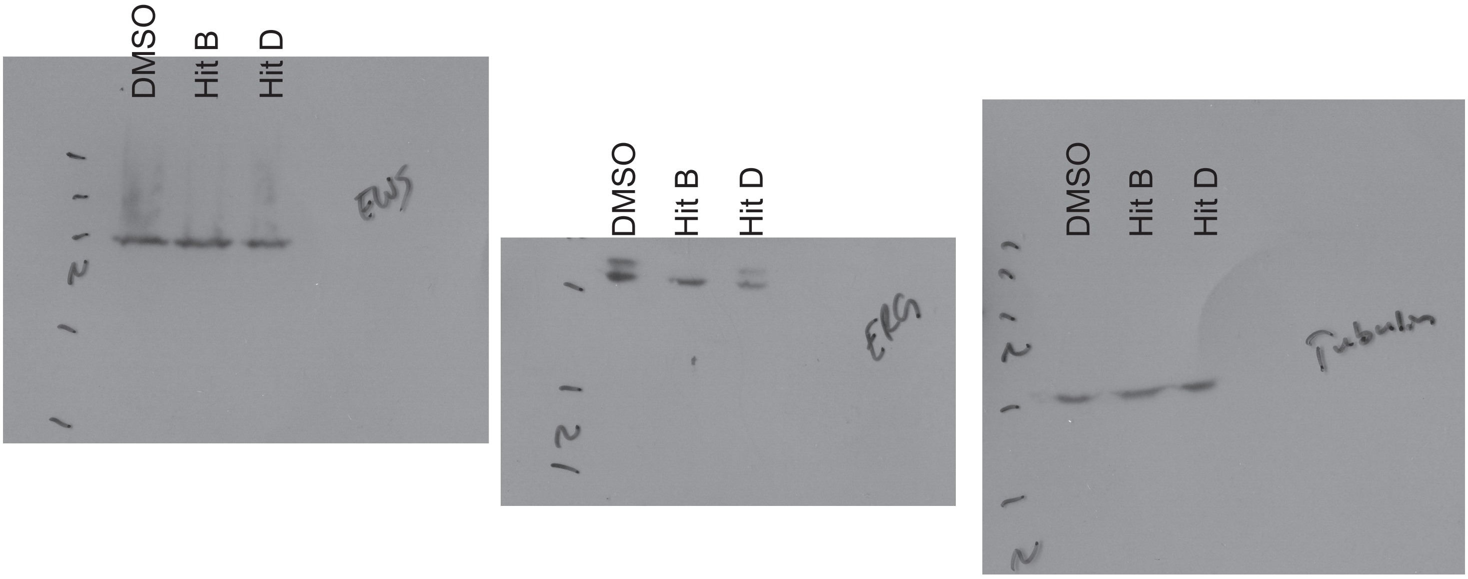

Supplement: S1 File — (PDF) [file pone.0238999.s001.pdf]
